# Accuracy and reliability of a low-cost, handheld 3D imaging system for child anthropometry

**DOI:** 10.1371/journal.pone.0205320

**Published:** 2018-10-24

**Authors:** Joel Conkle, Parminder S. Suchdev, Eugene Alexander, Rafael Flores-Ayala, Usha Ramakrishnan, Reynaldo Martorell

**Affiliations:** 1 Nutrition and Health Sciences Program, Laney Graduate School, Emory University, Atlanta, GA, United States of America; 2 Hubert Department of Global Health, Rollins School of Public Health, Emory University, Atlanta, GA, United States of America; 3 Division of Nutrition, Physical Activity and Obesity, National Center for Chronic Disease Prevention and Health Promotion, U.S. Centers for Disease Control and Prevention, Atlanta, GA, United States of America; 4 Department of Pediatrics, School of Medicine, Emory University, Atlanta, GA, United States of America; 5 Body Surface Translations, Inc. (BST), Athens, GA, United States of America; Institut de recherche pour le developpement, FRANCE

## Abstract

The usefulness of anthropometry to define childhood malnutrition is undermined by poor measurement quality, which led to calls for new measurement approaches. We evaluated the ability of a 3D imaging system to correctly measure child stature (length or height), head circumference and arm circumference. In 2016–7 we recruited and measured children at 20 facilities in and around metro Atlanta, Georgia, USA; including at daycare, higher education, religious, and medical facilities. We selected recruitment sites to reflect a generally representative population of Atlanta and to oversample newborns and children under two years of age. Using convenience sampling, a total of 474 children 0–5 years of age who were apparently healthy and who were present at the time of data collection were included in the analysis. Two anthropometrists each took repeated manual measures and repeated 3D scans of each child. We evaluated the reliability and accuracy of 3D scan-derived measurements against manual measurements. The mean child age was 26 months, and 48% of children were female. Based on reported race and ethnicity, the sample was 42% Black, 28% White, 8% Asian, 21% multiple races, other or race not reported; and 16% Hispanic. Measurement reliability of repeated 3D scans was within 1 mm of manual measurement reliability for stature, head circumference and arm circumference. We found systematic bias when analyzing accuracy—on average 3D imaging overestimated stature and head circumference by 6 mm and 3 mm respectively, and underestimated arm circumference by 2 mm. The 3D imaging system used in this study is reliable, low-cost, portable, and can handle movement; making it ideal for use in routine nutritional assessment. However, additional research, particularly on accuracy, and further development of the scanning and processing software is needed before making policy and clinical practice recommendations on the routine use of 3D imaging for child anthropometry.

## Introduction

Body measurement, or anthropometry, can be compared to a reference population to define nutritional status and to monitor child growth. Length or height, weight, and head circumference (HC) are common anthropometric measures for infants and children under 5 years of age. Anthropometry is used clinically to diagnose malnutrition [1–5], to identify underlying conditions [3], to assess risk for future disease [6, 7], and for clinical research [8]. At the population level, public health practitioners include anthropometry in research and surveys to identify causes and effects of abnormal nutritional status, to monitor trends through surveillance, and to target and evaluate interventions related to nutrition [7]. Anthropometry is also used to evaluate agricultural initiatives, and the global development community uses population-level anthropometry as an indicator of national economic development. Height-for-age is accepted as a more comprehensive indicator of poverty than income [9], and there is recognition that nutrition is essential for human capital development [10]. There is a target to improve stunting in the Sustainable Development Goals [11], and anthropometric indicators are used for allocation of Official Development Assistance [12].

Given that child growth has broad effects on health, nutrition, and development, it is important that anthropometric measurements are of high quality. Studies in primary care facilities of developed countries found that measurement error led to inaccurate and unreliable circumference measurement for adults [13, 14] and unreliable length and circumference measurements for children [15, 16]. There is also evidence that a lack of standardization and maintenance of anthropometric equipment in health facilities leads to misclassification of child weight status [17]. Three separate evaluations covering hundreds of large-scale, established surveys in developing countries found that on average more than 3% of weight or height measurements were biologically implausible [18–20]. According to a World Health Organization (WHO) Expert Committee, when more than one percent of measurements are considered biologically implausible, a survey is likely to be of poor quality [21].

The usefulness of anthropometry is undermined by poor measurement quality, which has led to calls for the use of technology to improve quality of child anthropometry [18, 22]. This study evaluated the ability of a portable, three-dimensional (3D) imaging system to accurately and reliably measure child stature (length or height), head circumference, and mid-upper arm circumference (MUAC).

## Subjects and methods

### Study design and participants

We designed the Body Imaging for Nutritional Assessment Study (BINA) to evaluate the accuracy and reliability of a 3D imaging system in comparison to manual measurements for child anthropometry. We chose to compare to manual measurement because growth standards are based on manual measurement, and when manual measurement is done well the levels of precision and accuracy are sufficient for nutritional assessment [23, 24]. The study was approved by the Emory Institutional Review Board (IRB), and included two phases. In the first phase we calibrated software to process 3D scans into measurements by scanning and measuring 36 children. In the second phase, the topic of this paper, we tested 3D imaging on a new sample of children. Children under five years of age who were apparently healthy and whose primary caregiver gave informed, written consent were eligible for the study. Caretakers received a nominal gift card ($15) for each child participating in the study. We recruited and measured children at 20 facilities in and around metro Atlanta, GA, USA; including at daycare, higher education, religious, and medical facilities. We selected recruitment sites to reflect a generally representative population of Atlanta children and included a maternity ward to sample newborns. Daycare centers received gift cards for participating as a study site. We formed a convenience sample by recruiting children on-site, via email, and through facility administrative staff; recruitment was ongoing throughout data collection, which lasted from September 2016 to February 2017. The intended sample size for the study was set at 500, with a target sample size of 100 for each of the following age groups: 0–5 months, 6–11 months, 12–17 months, 18–23 months and 24–59 months. We did not carry out a-priori power calculations. We set sample size targets by age group to oversample children under two years of age, an age group that is particularly difficult to measure manually, and to allow for an assessment of variability of measurement error across the entire span of 0–4 years.

### Test methods

Five trained anthropometrists with post-secondary education performed all manual measurements and 3D scans. Anthropometrists received training over a three week period in August 2016 from expert anthropometrists at Emory University and passed a standardization test for manual anthropometry. Manual measurements followed the protocol used to develop the 2006 WHO Child Growth Standards (CGS) [25]; detailed methods for manual anthropometry in BINA are published elsewhere [23]. Staff from Body Surface Translations Inc. (BST) trained anthropometrists to take 3D scans in one day, and anthropometrists informally used 3D scanners throughout the three week training period to familiarize themselves with the technology. During the standardization test anthropometrists scanned children following study protocol, and after visual assessment we determined scans were of sufficient quality to proceed with the study.

**Fig 1:**
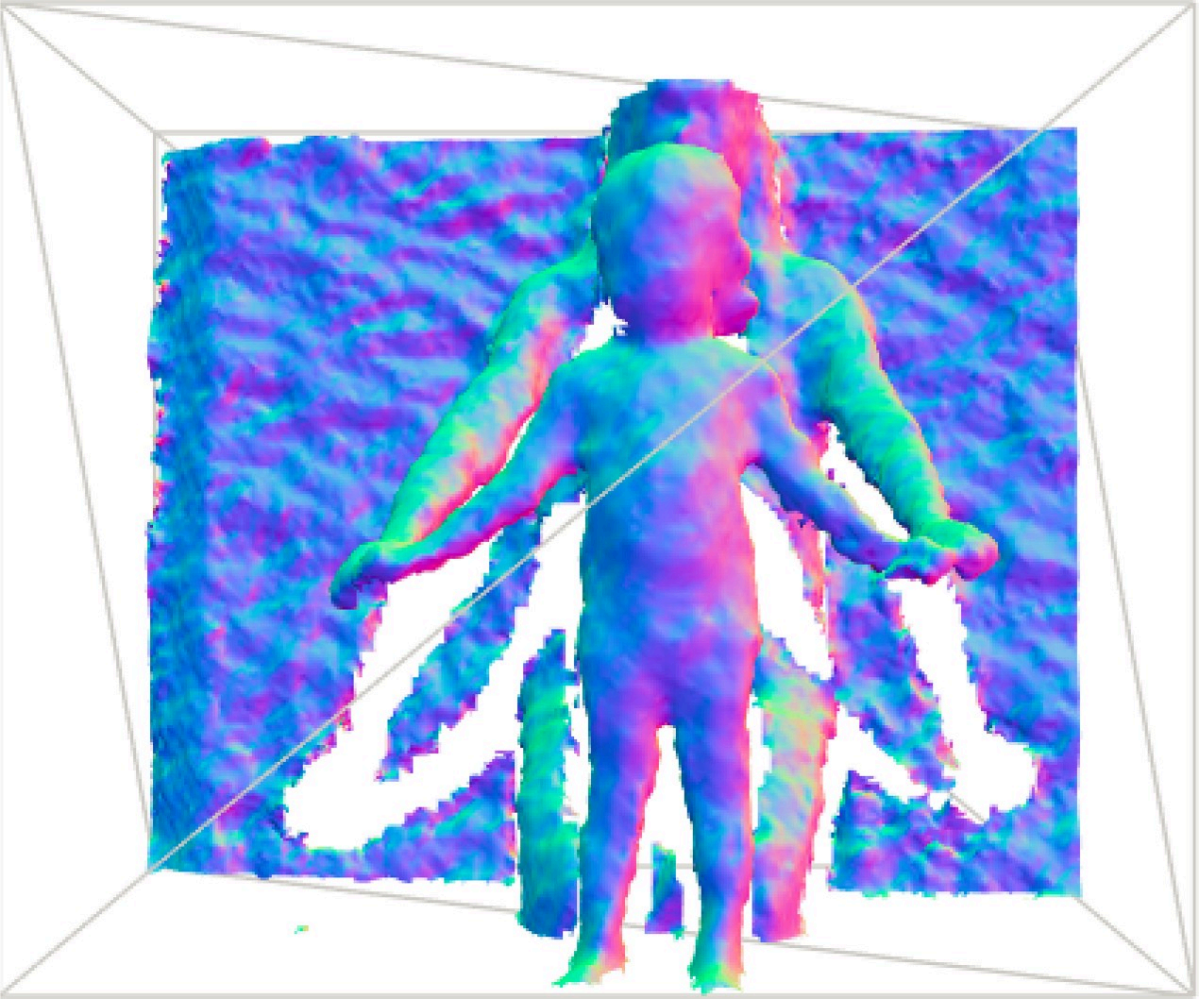
3D scan. Scan of child over two years of age with anthropometrist kneeling in the background. Scan as it appears to anthropometrist during data collection and before processing.

Each anthropometrist carried a 3D scanning device: a tablet with attached Structure Sensor 3D scanner (Occipital, San Francisco, CA, USA) and custom software from BST, AutoAnthro, for scanning and data entry of demographic information and manual measurements. AutoAnthro will be commercially available from BST. The 3D scanner we used was off-the-shelf, commercial hardware; and it was a fraction of the price of other scanners (USD $379). The scanner uses a Class 1 laser, which does not cause eye injury, and is the same type of laser used in video game technology. We collected scans (Fig 1) and then manual measurements consecutively at the same time of the day, usually in the morning. Each individual 3D imaging session comprised six scans, with three scans of the front of the child and three of the back. The software was designed for automated processing of six scans into body measurements. Consistent with manual anthropometry procedures, we scanned children two years of age and over standing up, and instructed younger children to lie down (S1–S3 Figs). Each child was scanned and measured twice by two different people, resulting in four sessions of scans and four sessions of manual measurements per child. Multiple measurements allowed analysis of both inter- and intra-measurer reliability.

### Analysis methods

In this study, one anthropometrist could be triggered to take a third measurement for manual measurements based on maximum allowable difference [23, 25], but not for scans. To determine a best-estimate from manual measurements, we excluded the outlying measurement in the case of a triggered, third measurement; and took the mean from the four remaining measurements (two from each anthropometrist). In this paper we refer to the average of four measurements as “best-estimate” and “all scan” for manual and scan-derived measurements respectively, and consider the former the reference standard. For analyzing reliability we limited our analysis to the first two manual measurements, ignoring any triggered third measurement; which provided a like-for-like comparison with scan-derived measurements. In the text we refer to the mean of two measurements as “repeated-manual” and “repeated-scan,” and to measurements derived from one measurement as “single-manual” and “single-scan”.

We used SPSS 20 (IBM Corp., Armonk, NY, USA) to test statistical significance of average bias with a two-sided, paired t-test with alpha of 0.05. Average bias is a metric of systematic bias. We also carried out Sign Tests—another metric of systematic bias that tests whether there were the same number of positive and negative differences using a Binomial Test.

Using StataSE 13’s (StataCorp, College Station, TX, USA) *baplot* module we created Bland-Altman (BA) Plots [26] to assess if accuracy remained constant across different child body sizes and to look at random bias. For the y-axis of the BA Plot we subtracted the best-estimate from the single-scan value, and for the x-axis we used the mean of single-scan and best-estimate. We used Pitman’s Test of Difference in Variance [27] to test the correlation between accuracy and the size of the child, and we calculated and plotted Limits of Agreement, which is the 95% precision interval for individual differences and is a metric of random bias. We disaggregated analysis based on age groups corresponding to a division in the estimation software, which used two anatomic models—one for children less than one month of age and another for children 1–59 months. If accuracy was not consistent across different sizes, indicated by a statistically significant Pitman’s Test, we carried out the additional step of regressing the difference on the independent, second single-scan as suggested by Bartlett and Frost to rule out difference in SD as the cause of a statistically significant Pitman’s Test [27]. We used Technical Error of Measurement (TEM) and the Coefficient of Reliability (R) as described by Ulijaszek [28] to measure reliability, which are the same measurements of reliability used to develop the WHO Child Growth Standards [25]. TEM represents one standard deviation and a 95% precision margin can be calculated by multiplying TEM by two. R measures the strength of correlation [28]. We used SPSS 20 to calculate the Intraclass Correlation Coefficient based on absolute agreement, which is another measurement of correlation that is familiar to a wider audience. Additional details of test and analysis methods are included in Methods of the supplementary online content.

## Results

### Participation and sample characteristics

S4 Fig shows the flow of participants in the study. We received informed consent for 555 children, of which 26 children were either not present or had aged out by the day of data collection. Of the remaining 529, we excluded 55 due to: refusal to be measured (n = 18), incomplete measurements (n = 8), health status (n = 5), loss of data due to technical errors during upload (upload software since corrected) (n = 10), and use of child in calibration of the 3D imaging system (n = 14); resulting in a final sample size of 474.

Table 1 presents sample characteristics. There was a low prevalence of wasting, stunting, underweight and overweight. The mean child age was 26 months and 48% of children were female. Based on reported race and ethnicity, the sample was 42% Black, 28% White, 8% Asian, 21% Multiple Races, Other or Race Not Reported; and 16% Hispanic. Children under two years of age and newborns were overrepresented, and nearly all of the newborns were less than four days old.

**Table 1:** Sample characteristics.

Age in months, mean (range)		25.7	(0–59)
Age Groups, no. (%)			
	Newborn (<1 month)	82	(17%)
	1–11.9 months	66	(14%)
	1–1.9 years	75	(16%)
	2–2.9 years	85	(18%)
	3–4.9 years	166	(35%)
Sex, no. (%)			
	Female	228	(48%)
Race, no. (%)			
	Black	201	(42%)
	White	134	(28%)
	Asian	40	(8%)
	Multiple, Other or Not Reported	99	(21%)
Ethnicity, no. (%)			
	Non-Hispanic	385	(81%)
	Hispanic	77	(16%)
	Not Reported	12	(3%)
Anthropometric Indices, mean, SD			
	Weight-for-Age Z-score (WAZ)	0.06	1.04
	Height-for-Age Z-score (HAZ)	-0.29	1.07
	Weight-for-Height Z-score (WHZ)	0.34	0.92
	Head Circumference Z-Score (HCZ)	0.24	1.02
	Arm Circumference Z-Score (ACZ)	0.78	0.94
Nutritional Status, no. (%)			
	Underweight (<-2 SD WAZ)	11	(2.3%)
	Stunted (<-2 SD HAZ)	21	(4.4%)
	Wasted (<-2 SD WHZ)	2	(0.4%)
	Overweight (>2 SD WHZ)	22	(4.7%)

### Accuracy

When using all-scan, the average bias of scan-derived measurements in cm was +0.6 (95% confidence interval (CI): 0.56, 0.62) for stature, +0.3 (CI: 0.30, 0.34) for HC, and -0.2 (CI: -0.21, -0.17) for MUAC (S1 Table). Differences were consistent and statistically significant at p < .0001 whether measurements were derived from single-scan, repeated-scan, or all-scan. However, the number of scan sessions did have an effect on the spread of differences and repeated measurements reduced variance as expected. For stature 97% of all-scan measurements were higher than manual measurements, or positive, and the 95% limit of agreement (LoA) showed that 95% of individual differences were within -0.1 to 1.2 cm; single-scan measurements were 78% positive with a LoA of -0.7 to 1.9 cm.

**Fig 2:**
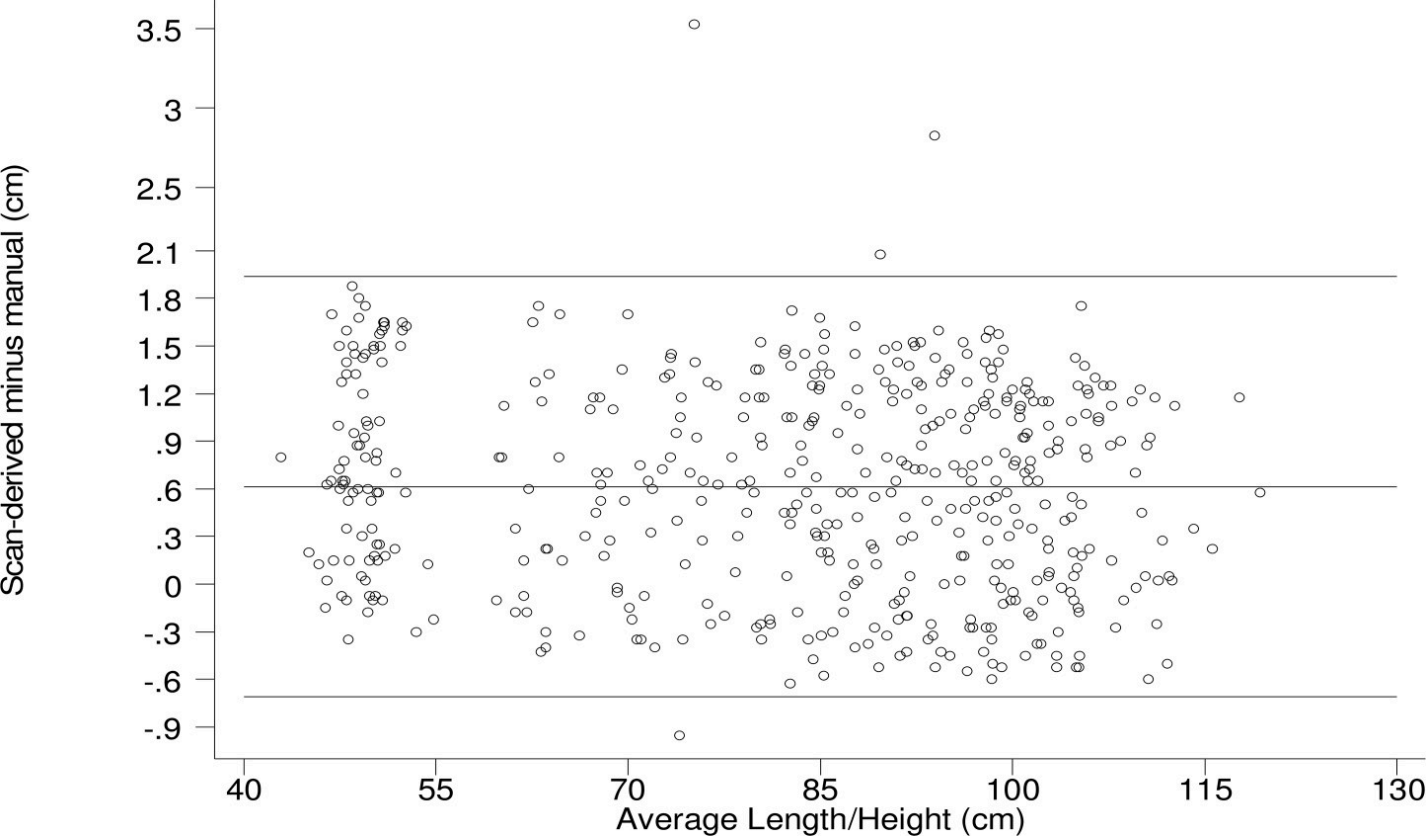
Bland-Altman plot. Length/height best-estimate manual measurement subtracted from single-scan measurement (y-axis) plotted against average based on both measurement types (x-axis) among children 0–59 months of age.

We visually inspected the accuracy of scan-derived measurements using Bland-Altman Plots (Fig 2). Compared to children 1–59 months of age 3D imaging was less accurate for newborns for all measures (Table 2). After disaggregating by age group (corresponding to the two anatomic models) Pitman’s Test was not significant for stature and HC, indicating no differential accuracy by size within the two age groups. For MUAC, Pitman’s Test was statistically significant (p < .01), suggesting differential accuracy by size within both age groups. Subsequent linear regression confirmed differential accuracy by size for MUAC— 3D imaging was less accurate for children with smaller MUAC. After separating children 1–59 months of age into quintiles based on MUAC, average bias of scan-derived measurements in cm was -0.31 (MUAC 9.6–15.1 cm), -0.18 (MUAC 15.1–16.0 cm), -0.15 (MUAC 16.0–16.7 cm), -0.02 (MUAC 16.7–17.6 cm), and -0.05 (MUAC 17.6–25.3 cm).

**Table 2:** Statistics related to Bland-Altman Plots by age group.

Age group	Measurement	Measurement difference^a^ in cm, μ (95% CI)	95% limits of agreement in cm	Pitman’s Test		n
				r	p-value	
<1 month	Length/height	0.825 (0.689 to 0.961)	-0.412 to 2.062	0.188	0.091	82
	Head circumference	0.553 (0.464 to 0.642)	-0.261 to 1.367	0.132	0.237	82
	Arm circumference	-0.437 (-0.516 to -0.359)	-1.149 to 0.274	0.291	0.008	82
1–59 months	Length/height	0.571 (0.505 to 0.636)	-0.756 to 1.897	-0.005	0.919	392
	Head circumference	0.262 (0.218 to 0.306)	-0.616 to 1.140	-0.044	0.386	392
	Arm circumference	-0.142 (-0.180 to -0.105)	-0.893 to 0.608	0.259	0.000	392

^a^ Single scan measurement minus best-estimate manual measurement

Among children 1–59 months of age there were no statistically significant or meaningful differences in accuracy by race or hairstyle (S2 Table). The largest difference was a 0.04 cm difference in average bias for head circumference between Black and White children.

### Reliability

**Fig 3:**
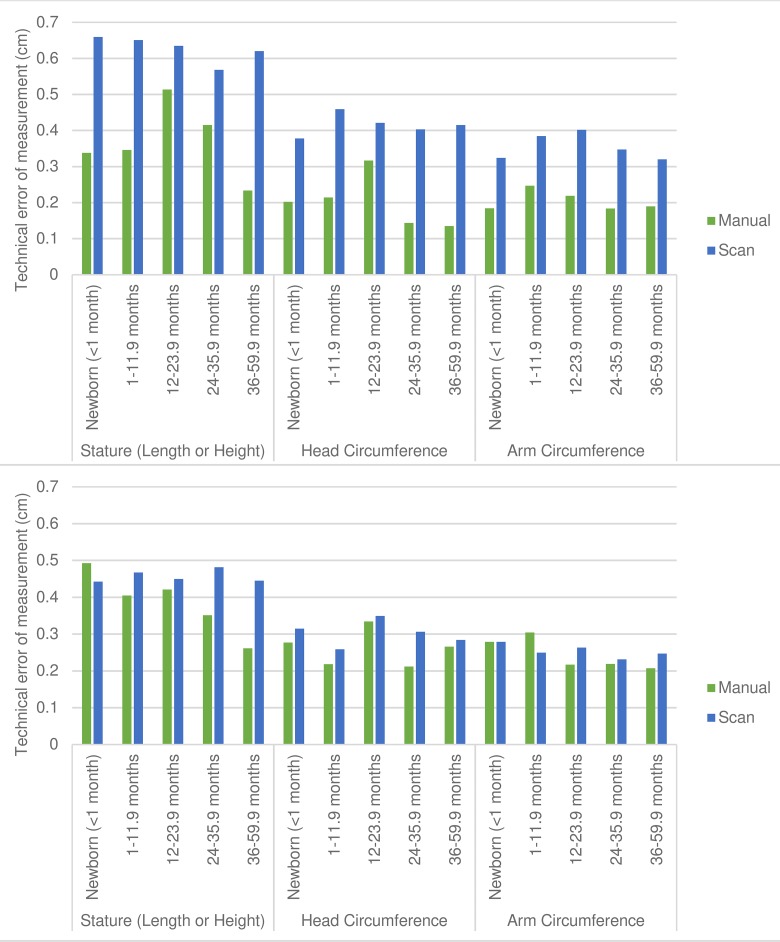
Intra- and inter-observer technical error of measurement (TEM). Scan-derived (light bars) versus manual measurement (dark bars) intra-observer TEM (A) and inter-observer TEM (B) for stature, head circumference and arm circumference disaggregated by age group. Inter-observer TEM based on average of repeated measures and intra-observer TEM based on single measures.

The intra-observer TEM for stature among children of all ages was 0.62 cm for scan-derived measurements, indicating that for a single observer the second scan-derived stature was within ±0.62 cm of the first scan-derived stature for two out of three children, and that for 95% of children the difference was within ±1.2 cm (Fig 3A and S3 Table). Manual measurement intra-observer TEM for stature among children of all ages was within ±0.72 cm for 95% of children. Intra-observer TEM from scan-derived measurements was higher than that from manual measurements for all measures and across all age groups, but unlike manual measurements, there were no meaningful differences by age group for scan-derived measurements (Fig 3A).

**Fig 4:**
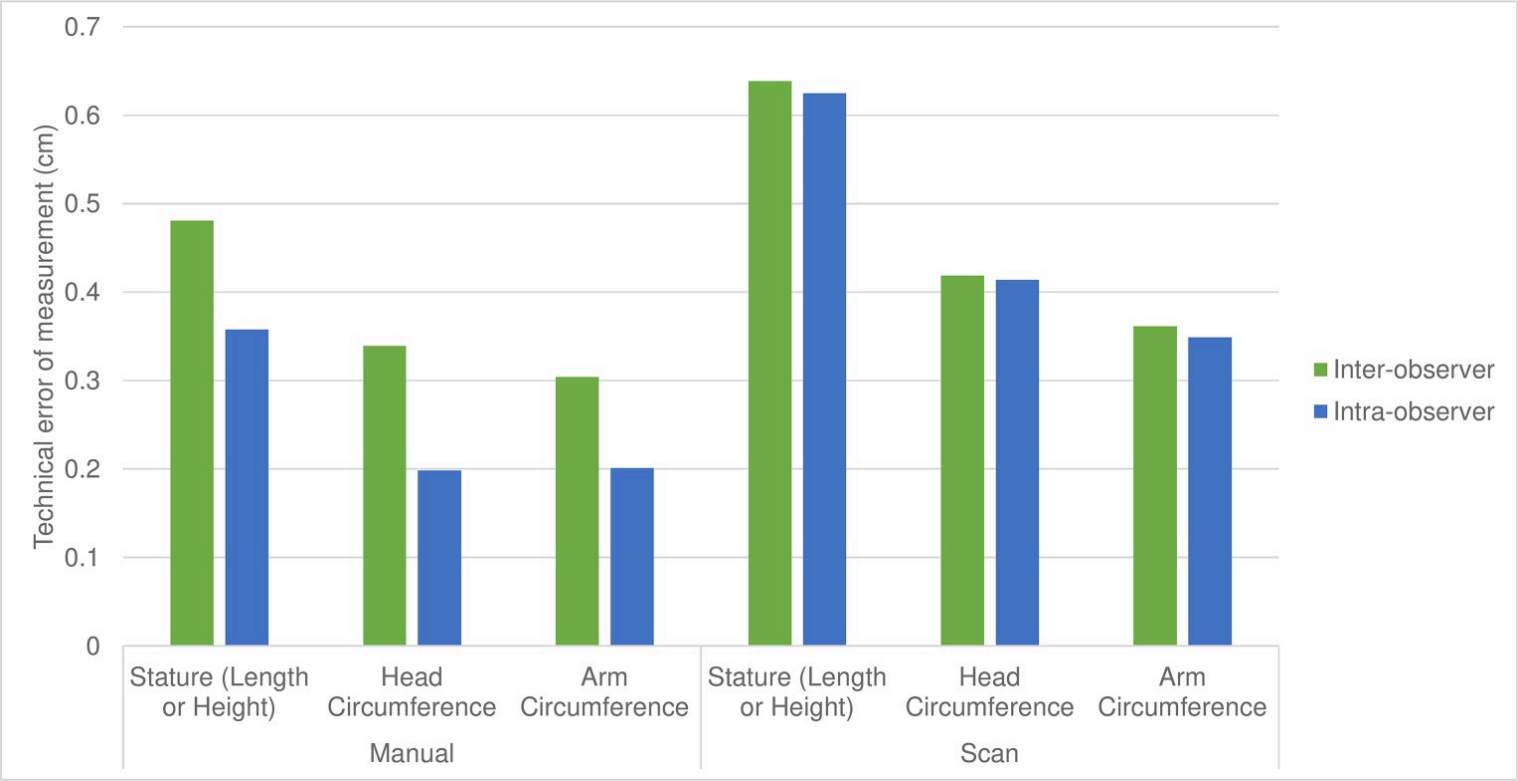
Single measure intra- and inter-observer technical error of measurement (TEM). Inter-observer TEM (dark bars) versus intra-observer TEM (light bars) for scan-derived (right) and manual measurements (left). Both inter- and intra-observer TEM based on single measures.

For all children under 5 years of age inter-observer TEM from repeated scans was within 0.1 cm of TEM from repeated manual measurements for all measures (Fig 3B). We also examined inter-observer TEM based on single measurements. Single-scan inter-observer TEM was higher than single-manual inter-observer TEM (Fig 4).

When using single measurements inter-observer TEM was higher than intra-observer TEM for manual measurement, but not for scans (Fig 4), indicating that scanning produced similar results for anyone who repeated the scan. Total TEM combines the intra- and inter-observer TEM from S3 Table into a single metric. For manual measurements Total TEM was 0.51 cm, 0.33 cm, and 0.31 cm for stature, HC and MUAC respectively; compared to 0.77 cm, 0.51 cm, and 0.43 cm for scan-derived measurements.

The Coefficient of Reliability based on Total TEM was 1.00, 1.00, and 0.99 for stature, HC and MUAC respectively from manual measurements; and 1.00, 0.99, and 0.98 for scan-derived measurements. The high R indicates excellent agreement for repeated measurements. Intraclass correlation coefficients, another measure of agreement, were also close to 1.00 for intra- and inter-observer repeated measurements (S3 Table), confirming the excellent correlation between repeated measurements for both manual and scan-derived measurements.

## Discussion

We previously demonstrated that BINA collected gold-standard, manual anthropometry based on analysis of biological plausibility, reliability, and z-score standard deviations [23]. In this paper we compared measurements derived from 3D imaging to these gold-standard, manual measurements. For biological plausibility, 3D imaging and manual measurement were exactly the same, with both methods producing plausible measurements >99% of the time; this finding indicates acceptable quality based on WHO expert committee criteria for biological plausibility [21]. We also found that repeated-scan 3D imaging produced measurement reliability that was within 1 mm of manual measurement reliability for stature, HC and MUAC; this level of reliability puts 3D imaging on par with manual measurements collected in the Multicenter Growth Reference Study (MGRS) used to develop the 2006 WHO CGS [24]. Considering only biological plausibility and reliability, 3D imaging performed as well as gold-standard manual measurements for child anthropometry. However, 3D imaging systematically underestimated or overestimated child size when compared to our best-estimate of size from manual measurement.

Before reaching any conclusion on the readiness of 3D imaging for child anthropometry, we would need to determine if the systematic inaccuracy found in this study is population specific. If the same under- and overestimation was found in a different sample with different anthropometrists, we could then identify and fix the cause of the bias in the model fit or simply build adjustments into the software. Knowing the cause of bias could facilitate adjustments. We hypothesized that inaccuracies in our study were the result of difficulty in manual measurement for MUAC, and not accounting for exact protocol of manual measurement in the design of scan processing software for head circumference and stature.

Research similar to BINA should be carried out, ideally in developed and low and middle income countries, to help answer questions on systematic inaccuracy and also to address some of the other limitations of our study. The 3D imaging system may perform differently under the harsher conditions of a household survey or community-based screening. Increased handling during transport, lack of access to electricity, lighting, dust, space constraints and other environmental factors could all affect the functionality of the 3D scanner.

Additional limitations to our study stem from sampling design and automated processing. The sample size was not specified during study design based on power calculations, and due to limited sample size and the choice of population we did not fully explore differences in prevalence estimates and did not analyze sensitivity and specificity for clinically significant indicators, such as obesity, wasting and severe stunting. In addition, findings from our non-random sample cannot be generalized to any specific age group, and the processing of 3D scans was not fully automated as originally planned. Anthropometrists took more scans than needed and manually selected the best quality scans. Also, the orientation (front/back) of each scan was manually coded. Further software development is needed to achieve full automation, which could improve repeatability.

Our primary interest in researching 3D imaging for child anthropometry was to improve the quality of anthropometric data, and while not conclusive, our findings suggest that 3D imaging could play a role in quality improvement. Compared to manual measurement, we spent substantially less time on training and supervision for 3D scanning, and achieved similar reliability. Also, our findings on scan-derived measurement reliability suggest that scanning was not affected by child age, which can be viewed as a proxy for cooperation, or anthropometrist’s technique. Both cooperation and measuring technique are known to negatively affect anthropometric data quality. Qualitative research on BINA anthropometrists’ experiences using 3D scanners is currently underway and this may help to provide additional evidence on the potential of 3D imaging to improve anthropometric data quality. However, our study was not designed to determine if 3D imaging led to better quality, and anthropometrists in BINA; who were well educated, highly motivated, and well-trained; achieved high quality anthropometric data with both 3D imaging and manual measurement. Conclusive evidence on quality improvement will not be available until 3D imaging is tested in a setting of poor quality manual measurement.

Results from our analysis of z-scores and classification (S4 and S5 Tables); along with an expanded discussion on reliability, bias hypotheses and study limitations; is included in the supplementary online content.

## Conclusions

3D imaging is not new for anthropometry [29–33], but the 3D scanner used in our study was inexpensive, brought unique functionality, and shows promise as a substitute for traditional anthropometry measurements. The scanning device is small, lightweight, and the software developed by BST only requires a series of snapshots, which allows some subject movement. The 3D imaging system used in our study, AutoAnthro, could be an ideal replacement for bulky height boards used in surveys, and to our knowledge it is the first portable 3D system specifically designed for whole body scanning of infants and young children. In conclusion, our findings indicate that AutoAnthro can produce reliable child anthropometry, but further research and development is needed before 3D imaging can be recommended as a solution to improving the quality of anthropometric data.

## Supporting information

S1 Fig 3D scan arm poses. Poses for children two years of age and over.
(TIF)

S2 Fig 3D scan measurement points. Points (in black) selected on base model to measure head and arm circumference.
(TIF)

S3 Fig The basic fitting process. Scan data is in green, articulated model surface in red, “bones” and “joints” in blue. On the left, the initial size and pose of model relative to data. On the right, the model has been automatically sized and posed to fit the scan data.
(TIF)

S4 Fig Flow of study participants. (TIF)

S1 Table Accuracy of scan-derived measurements. Comparison to best-estimate, manual measurements among all children under five years of age.
(DOCX)

S2 Table Accuracy by race and hairstyle. Considering best-estimate manual measurements and scan-derived measurements from all sessions among children 1 to 59.9 months of age.
(DOCX)

S3 Table Intra-observer reliability and inter-observer reliability. Based on repeated manual measurements and repeated scan sessions by age group.
(DOCX)

S4 Table Z-score mean, standard deviation (SD) and prevalence by selected z-score-for-age cutoffs. Among children 1–59.9 months of age.
(DOCX)

S5 Table Sensitivity and specificity of adjusted, scan-derived measures. Comparison to best-estimate manual measures among children 1–59.9 months of age.
(DOCX)

S1 Text Supplementary methods, results and discussion. (DOCX)
