# Supplementary figures and images for: Accuracy and reliability of a low-cost, handheld 3D imaging system for child anthropometry
Source: PLoS One. 2018 Oct 24;13(10):e0205320. doi: 10.1371/journal.pone.0205320 (PMC6200231; doi:10.1371/journal.pone.0205320)

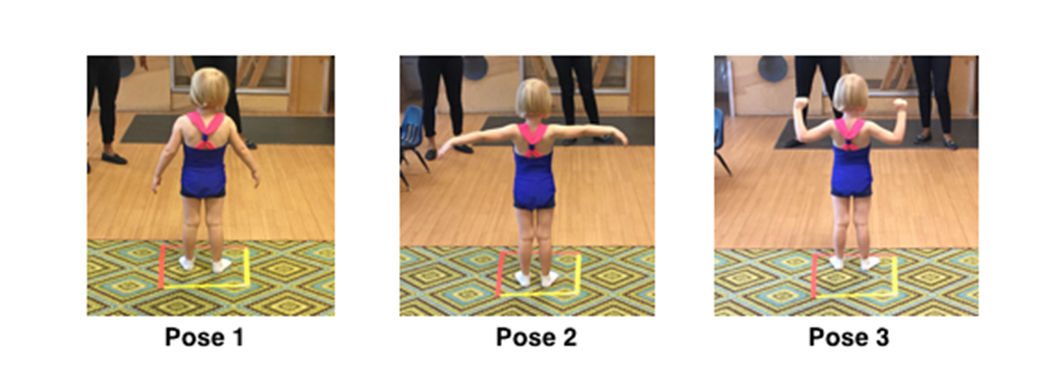

Supplement: S1 Fig — Poses for children two years of age and over. (TIF) [file pone.0205320.s001.tif]

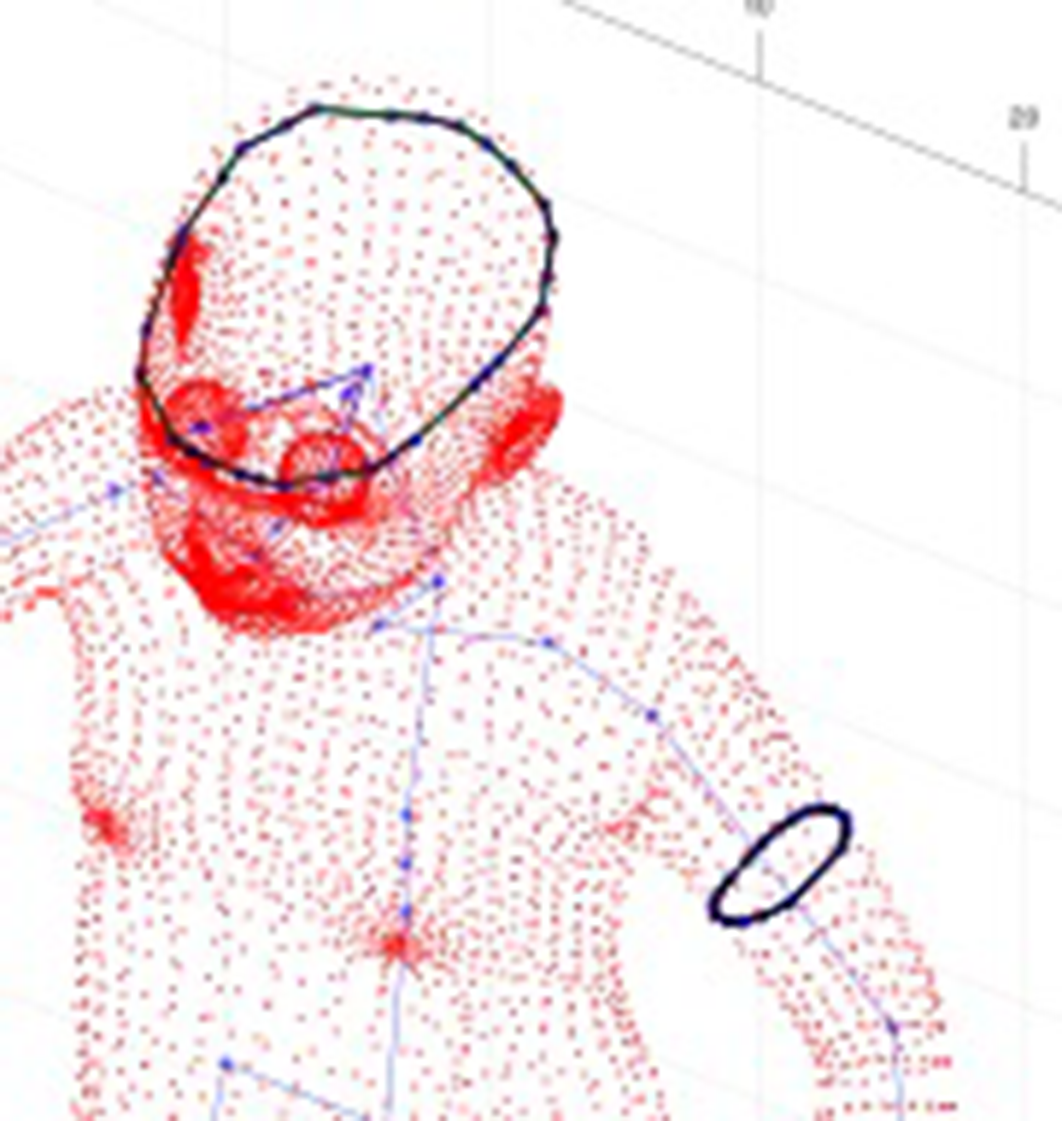

Supplement: S2 Fig — Points (in black) selected on base model to measure head and arm circumference. (TIF) [file pone.0205320.s002.tif]

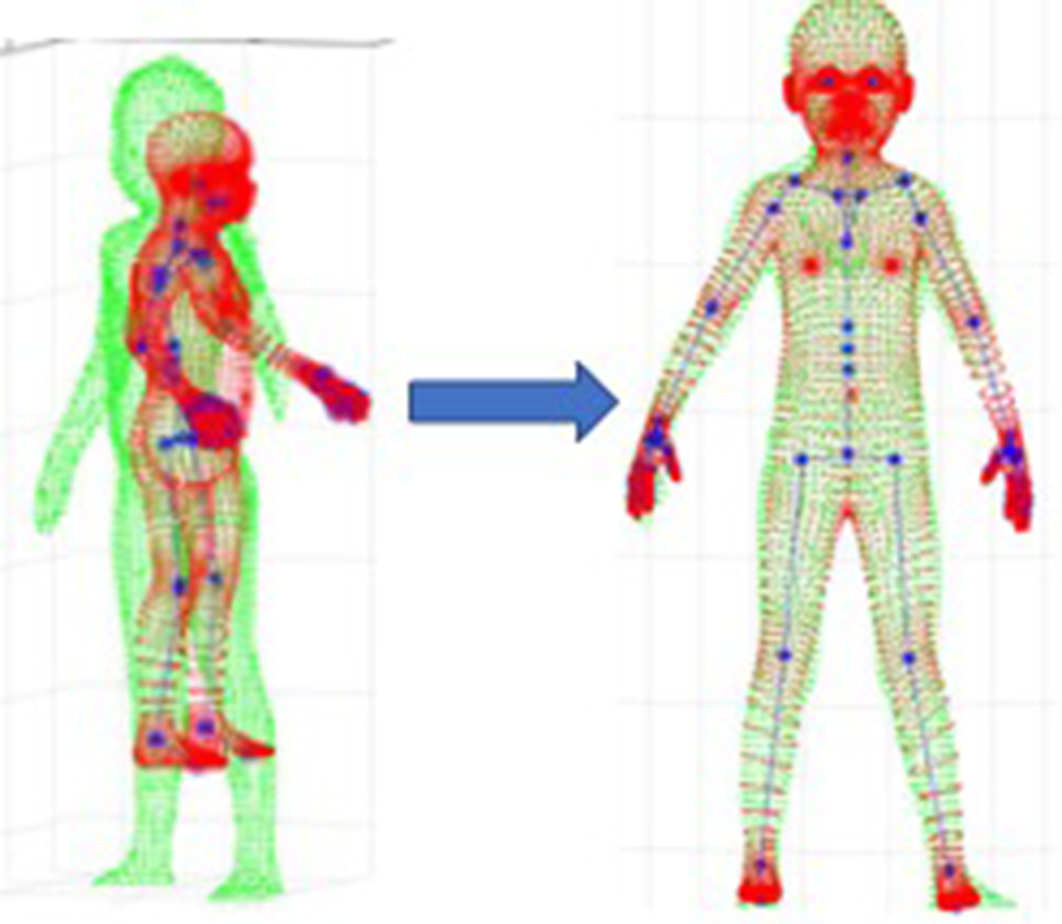

Supplement: S3 Fig — Scan data is in green, articulated model surface in red, “bones” and “joints” in blue. On the left, the initial size and pose of model relative to data. On the right, the model has been automatically sized and posed to fit the scan data. (TIF) [file pone.0205320.s003.tif]

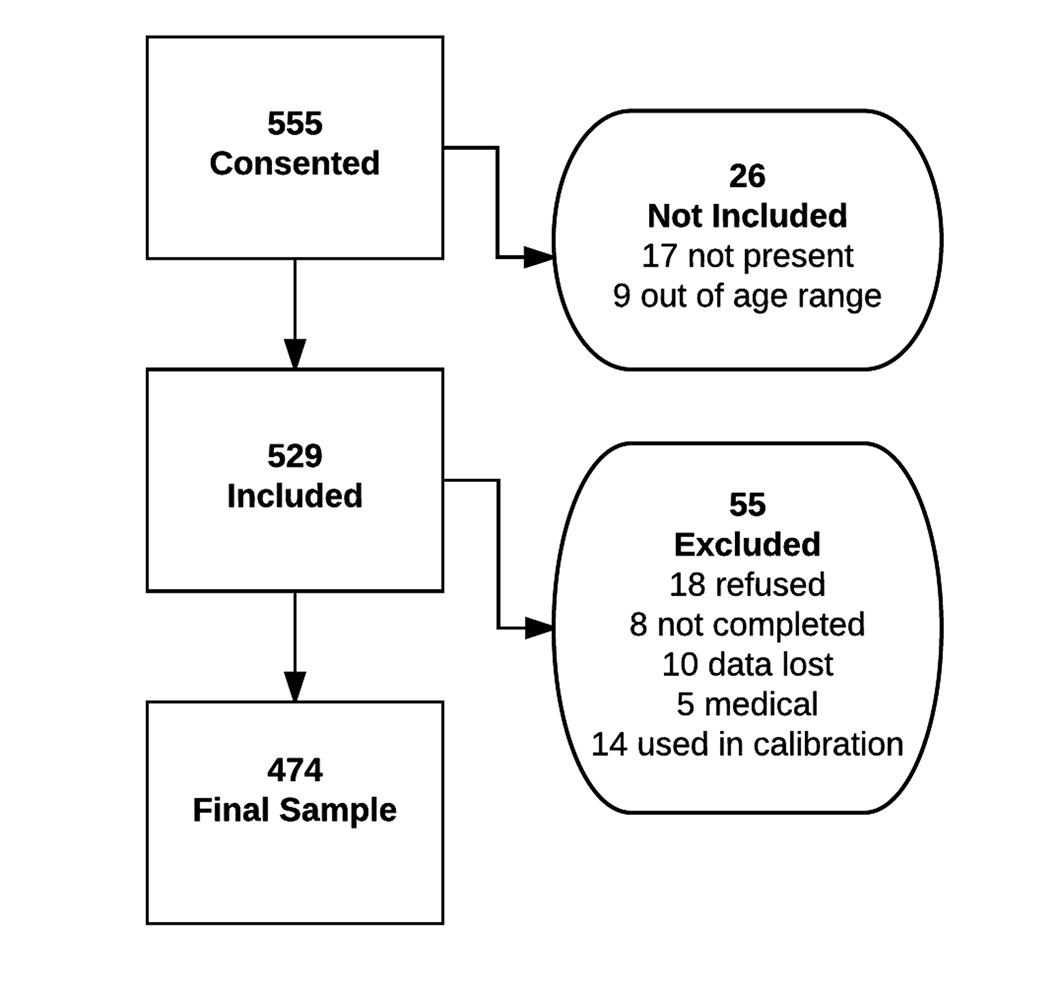

Supplement: S4 Fig — (TIF) [file pone.0205320.s004.tif]
