# Supplementary material for: Accuracy and reliability of a low-cost, handheld 3D imaging system for child anthropometry
Source: PLoS One. 2018 Oct 24;13(10):e0205320. doi: 10.1371/journal.pone.0205320 (PMC6200231; doi:10.1371/journal.pone.0205320)
Supplement: S1 Table — Comparison to best-estimate, manual measurements among all children under five years of age. (DOCX) [file pone.0205320.s005.docx]

|  |  |  | **Paired T-Test** | | | | | **Percent of Positive Differences^c^** |
| --- | --- | --- | --- | --- | --- | --- | --- | --- |
|  |  | **Measurer and Observation^a^** | **Mean from Scan** | **Mean Difference (Scan-Manual)** | **T value for Difference From 0^b^** | **Difference 95% Limits of Agreement** | |  |
|  |  |  |  |  |  | **Lower Limit** | **Upper Limit** |  |
| Stature (Length or Height) | | | | | | | | |
|  | Single scan session | | | | | | | |
|  |  | Measurer 1 (M1) Observation 1 (O1) | 82.9 | 0.61 | 20.2 | -0.7 | 1.9 | 78% |
|  |  | M1, Observation 2 (O2) | 82.9 | 0.56 | 19.2 | -0.7 | 1.8 | 78% |
|  |  | Measurer 2 (M2), O1 | 82.9 | 0.58 | 20.1 | -0.7 | 1.8 | 80% |
|  |  | M2, O2 | 82.9 | 0.60 | 21.1 | -0.6 | 1.8 | 80% |
|  | Average from two scan sessions | | | | | | | |
|  |  | M1 | 82.9 | 0.59 | 26.8 | -0.4 | 1.5 | 90% |
|  |  | M2 | 82.9 | 0.59 | 29.2 | -0.3 | 1.5 | 91% |
|  | Average from four scan sessions | | | | | | | |
|  |  | M1&M2 |  |  |  |  |  |  |
|  |  | M1&M2 | 82.9 | 0.59 | 39.2 | -0.1 | 1.2 | 97% |
| Head Circumference | | | | | | | | |
|  | Single scan session | | | | | | | |
|  |  | M1, O1 | 46.1 | 0.31 | 15.2 | -0.6 | 1.2 | 72% |
|  |  | M1, O2 | 46.1 | 0.32 | 16.9 | -0.5 | 1.2 | 73% |
|  |  | M2, O1 | 46.1 | 0.32 | 16.7 | -0.5 | 1.1 | 77% |
|  |  | M2, O2 | 46.1 | 0.34 | 16.4 | -0.6 | 1.2 | 73% |
|  | Average from two scan sessions | | | | | | | |
|  |  | M1 | 46.1 | 0.32 | 22.1 | -0.3 | 0.9 | 83% |
|  |  | M2 | 46.1 | 0.33 | 22.1 | -0.3 | 1.0 | 84% |
|  | Average from four scan sessions | | | | | | | |
|  |  | M1 & M2 | 46.1 | 0.32 | 29.9 | -0.1 | 0.8 | 93% |
| Arm Circumference | | | | | | | | |
|  | Single scan session | | | | | | | |
|  |  | M1, O1 | 15.2 | -0.19 | -10.9 | -1.0 | 0.6 | 34% |
|  |  | M1, O2 | 15.2 | -0.20 | -12.4 | -0.9 | 0.5 | 35% |
|  |  | M2, O1 | 15.2 | -0.20 | -11.7 | -0.9 | 0.5 | 33% |
|  |  | M2, O2 | 15.2 | -0.17 | -10.1 | -0.9 | 0.6 | 36% |
|  | Average from two scan sessions | | | | | | | |
|  |  | M1 | 15.2 | -0.20 | -15.6 | -0.7 | 0.4 | 25% |
|  |  | M2 | 15.2 | -0.19 | -14.5 | -0.7 | 0.4 | 28% |
|  | Average from four scan sessions | | | | | | | |
|  |  | M1 & M2 | 15.2 | -0.19 | -19.7 | -0.6 | 0.2 | 20% |
| ^a^ Sample size was 474 paired observations for all rows: single scan, average of two scans and average from four scans.  ^b^All mean differences significantly different from zero at p<.0001.  ^c^From binomial test percentages are all significantly different from 50% at p<.0001. | | | | | | | | |
