# Supplementary material for: Accuracy and reliability of a low-cost, handheld 3D imaging system for child anthropometry
Source: PLoS One. 2018 Oct 24;13(10):e0205320. doi: 10.1371/journal.pone.0205320 (PMC6200231; doi:10.1371/journal.pone.0205320)
Supplement: S2 Table — Considering best-estimate manual measurements and scan-derived measurements from all sessions among children 1 to 59.9 months of age. (DOCX) [file pone.0205320.s006.docx]

|  | **Race or Hairstyle** | **n** | **Mean Difference** (scan-manual) | **Standard Deviation (SD)** | **One-Way Anyalysis of Variance** | |
| --- | --- | --- | --- | --- | --- | --- |
|  |  |  |  |  | F | Significance |
| Stature by Race | | |  |  | 0.30 | 0.74 |
|  | Black | 136 | 0.53 | 0.33 |  |  |
|  | White | 126 | 0.55 | 0.32 |  |  |
|  | Other | 130 | 0.56 | 0.31 |  |  |
| Head Circumference by Race | | | |  | 1.37^a^ | 0.25 |
|  | Black | 136 | 0.30 | 0.24 |  |  |
|  | White | 126 | 0.26 | 0.17 |  |  |
|  | Other | 130 | 0.27 | 0.22 |  |  |
| Head Circumference by Hairstyle | | | |  | 0.39 | 0.53 |
|  | Large hair | 347 | 0.28 | 0.22 |  |  |
|  | Not large hair | 45 | 0.26 | 0.21 |  |  |
| Arm Circumference by Race | | | |  | 0.02 | 0.98 |
|  | Black | 136 | -0.15 | 0.20 |  |  |
|  | White | 126 | -0.15 | 0.18 |  |  |
|  | Other | 130 | -0.14 | 0.19 |  |  |
| ^a^Asymptomatically F distributed and tested with Brown-Forsythe | | | | | | |
