# Supplementary material for: Accuracy and reliability of a low-cost, handheld 3D imaging system for child anthropometry
Source: PLoS One. 2018 Oct 24;13(10):e0205320. doi: 10.1371/journal.pone.0205320 (PMC6200231; doi:10.1371/journal.pone.0205320)
Supplement: S3 Table — Based on repeated manual measurements and repeated scan sessions by age group. (DOCX) [file pone.0205320.s007.docx]

| **Intra-observer Reliability** | | | | | | | | | | | |
| --- | --- | --- | --- | --- | --- | --- | --- | --- | --- | --- | --- |
|  | **Sample Size** | **Average (cm)** | | **Mean Absolute Difference (cm)** | | **Technical Error of Measurement (TEM) (cm)** | | **Relative TEM (%TEM)** | | **Intraclass Correlation Coefficient (ICC)** | |
| **Row Labels** |  | **Manual** | **Scan** | **Manual** | **Scan** | **Manual** | **Scan** | **Manual** | **Scan** | **Manual** | **Scan** |
| Stature (Length or Height) |  |  |  |  |  |  |  |  |  |  |  |
| All (0-4.9 years) | 948 | 82.3 | 82.9 | 0.3 | 0.7 | 0.36 | 0.62 | 0.4 | 0.8 | 1.00 | 1.00 |
| Newborn (<1 month) | 164 | 48.8 | 49.6 | 0.3 | 0.8 | 0.34 | 0.66 | 0.7 | 1.3 | 0.96 | 0.86 |
| 1-11.9 months | 132 | 66.2 | 66.8 | 0.4 | 0.8 | 0.35 | 0.65 | 0.5 | 1.0 | 1.00 | 0.99 |
| 12-23.9 months | 150 | 81.2 | 81.8 | 0.4 | 0.7 | 0.51 | 0.63 | 0.6 | 0.8 | 0.99 | 0.98 |
| 24-35.9 months | 170 | 90.3 | 90.8 | 0.3 | 0.7 | 0.41 | 0.57 | 0.5 | 0.6 | 0.99 | 0.98 |
| 36-59.9 months | 332 | 101.7 | 102.2 | 0.2 | 0.7 | 0.23 | 0.62 | 0.2 | 0.6 | 1.00 | 0.99 |
| Head Circumference |  |  |  |  |  |  |  |  |  |  |  |
| All (0-4.9 years) | 948 | 45.7 | 46.1 | 0.2 | 0.5 | 0.20 | 0.41 | 0.4 | 0.9 | 1.00 | 1.00 |
| Newborn (<1 month) | 164 | 34.0 | 34.6 | 0.2 | 0.4 | 0.20 | 0.38 | 0.6 | 1.1 | 0.97 | 0.89 |
| 1-11.9 months | 132 | 43.1 | 43.5 | 0.2 | 0.5 | 0.21 | 0.46 | 0.5 | 1.1 | 0.99 | 0.97 |
| 12-23.9 months | 150 | 47.5 | 47.8 | 0.2 | 0.5 | 0.32 | 0.42 | 0.7 | 0.9 | 0.96 | 0.94 |
| 24-35.9 months | 170 | 48.8 | 49.0 | 0.1 | 0.5 | 0.14 | 0.40 | 0.3 | 0.8 | 0.99 | 0.94 |
| 36-59.9 months | 332 | 50.2 | 50.5 | 0.1 | 0.5 | 0.13 | 0.41 | 0.3 | 0.8 | 0.99 | 0.93 |
| Arm Circumference |  |  |  |  |  |  |  |  |  |  |  |
| All (0-4.9 years) | 948 | 15.4 | 15.2 | 0.2 | 0.4 | 0.20 | 0.35 | 1.3 | 2.3 | 0.99 | 0.99 |
| Newborn (<1 month) | 164 | 10.7 | 10.3 | 0.2 | 0.4 | 0.18 | 0.32 | 1.7 | 3.2 | 0.95 | 0.88 |
| 1-11.9 months | 132 | 14.7 | 14.4 | 0.2 | 0.4 | 0.25 | 0.38 | 1.7 | 2.7 | 0.98 | 0.95 |
| 12-23.9 months | 150 | 15.9 | 15.8 | 0.2 | 0.5 | 0.22 | 0.40 | 1.4 | 2.5 | 0.97 | 0.91 |
| 24-35.9 months | 170 | 16.6 | 16.5 | 0.2 | 0.0 | 0.18 | 0.35 | 1.1 | 2.1 | 0.98 | 0.93 |
| 36-59.9 months | 332 | 17.2 | 17.1 | 0.2 | 0.3 | 0.19 | 0.32 | 1.1 | 1.9 | 0.99 | 0.96 |
| **Inter-observer Reliability (average of repeated measures)** | | | | | | | | | | | |
|  | **Sample Size** | **Average in cm** | | **Mean Absolute Difference (cm)** | | **Technical Error of Measurement (TEM)** | | **Relative TEM (%TEM)** | | **Intraclass Correlation Coefficient (ICC)** | |
| **Row Labels** | **manual** | **manual** | **scan** | **manual** | **scan** | **manual** | **scan** | **manual** | **scan** | **manual** | **scan** |
| Stature (Length or Height) |  |  |  |  |  |  |  |  |  |  |  |
| All (0-4.9 years) | 474 | 82.3 | 82.9 | 0.4 | 0.5 | 0.37 | 0.46 | 0.5 | 0.5 | 1.00 | 1.00 |
| Newborn (<1 month) | 82 | 48.8 | 49.6 | 0.5 | 0.5 | 0.49 | 0.44 | 1.0 | 0.9 | 0.92 | 0.93 |
| 1-11.9 months | 66 | 66.2 | 66.8 | 0.4 | 0.5 | 0.40 | 0.47 | 0.6 | 0.7 | 1.00 | 0.99 |
| 12-23.9 months | 75 | 81.2 | 81.8 | 0.4 | 0.5 | 0.42 | 0.45 | 0.5 | 0.5 | 0.99 | 0.99 |
| 24-35.9 months | 85 | 90.3 | 90.8 | 0.3 | 0.6 | 0.35 | 0.48 | 0.4 | 0.5 | 0.99 | 0.99 |
| 36-59.9 months | 166 | 101.7 | 102.2 | 0.3 | 0.5 | 0.26 | 0.44 | 0.3 | 0.4 | 1.00 | 0.99 |
| Head Circumference |  |  |  |  |  |  |  |  |  |  |  |
| All (0-4.9 years) | 474 | 45.7 | 46.1 | 0.3 | 0.4 | 0.26 | 0.30 | 0.6 | 0.7 | 1.00 | 1.00 |
| Newborn (<1 month) | 82 | 34.0 | 34.6 | 0.3 | 0.4 | 0.28 | 0.31 | 0.8 | 0.9 | 0.94 | 0.92 |
| 1-11.9 months | 66 | 43.1 | 43.5 | 0.2 | 0.3 | 0.22 | 0.26 | 0.5 | 0.6 | 0.99 | 0.99 |
| 12-23.9 months | 75 | 47.5 | 47.8 | 0.3 | 0.4 | 0.33 | 0.35 | 0.7 | 0.7 | 0.96 | 0.95 |
| 24-35.9 months | 85 | 48.8 | 49.0 | 0.2 | 0.4 | 0.21 | 0.31 | 0.4 | 0.6 | 0.98 | 0.96 |
| 36-59.9 months | 166 | 50.2 | 50.5 | 0.2 | 0.3 | 0.27 | 0.28 | 0.5 | 0.6 | 0.97 | 0.97 |
| Arm Circumference |  |  |  |  |  |  |  |  |  |  |  |
| All (0-4.9 years) | 474 | 15.4 | 15.2 | 0.3 | 0.3 | 0.24 | 0.25 | 1.6 | 1.7 | 0.99 | 0.99 |
| Newborn (<1 month) | 82 | 10.7 | 10.3 | 0.3 | 0.3 | 0.28 | 0.28 | 2.6 | 2.7 | 0.89 | 0.90 |
| 1-11.9 months | 66 | 14.7 | 14.4 | 0.3 | 0.3 | 0.30 | 0.25 | 2.1 | 1.7 | 0.96 | 0.98 |
| 12-23.9 months | 75 | 15.9 | 15.8 | 0.2 | 0.3 | 0.22 | 0.26 | 1.4 | 1.7 | 0.97 | 0.96 |
| 24-35.9 months | 85 | 16.6 | 16.5 | 0.2 | 0.3 | 0.22 | 0.23 | 1.3 | 1.4 | 0.97 | 0.97 |
| 36-59.9 months | 166 | 17.2 | 17.1 | 0.2 | 0.3 | 0.21 | 0.25 | 1.2 | 1.4 | 0.98 | 0.98 |
