# Supplementary material for: Accuracy and reliability of a low-cost, handheld 3D imaging system for child anthropometry
Source: PLoS One. 2018 Oct 24;13(10):e0205320. doi: 10.1371/journal.pone.0205320 (PMC6200231; doi:10.1371/journal.pone.0205320)
Supplement: S4 Table — Among children 1–59.9 months of age. (DOCX) [file pone.0205320.s008.docx]

| Row Labels | Sample Size | Z-score Mean | Z-score SD | Prevalence by z-score-for-age cutoff | |
| --- | --- | --- | --- | --- | --- |
| Stature |  |  |  | HAZ <-1 SD | HAZ <-2SD |
| Best-estimate Manual | 392 | -0.25 | 1.10 | 21.9 | 4.6 |
| Single Manual | 392 | -0.24 | 1.10 | 22.7 | 4.3 |
| Unadjusted Single Scan | 392 | -0.07 | 1.12 | 17.6 | 3.8 |
| Adjusted Single Scan | 392 | -0.25 | 1.13 | 23.5 | 5.4 |
| Adjusted Repeated Scan | 392 | -0.25 | 1.11 | 22.7 | 4.6 |
| Head Circumference |  |  |  | HCZ >1 SD | HCZ >2 SD |
| Best-estimate Manual | 392 | 0.34 | 1.02 | 27.3 | 3.3 |
| Single Manual | 392 | 0.34 | 1.04 | 27.3 | 3.8 |
| Unadjusted Single Scan | 392 | 0.53 | 1.07 | 32.9* | 8.4*** |
| Adjusted Single Scan | 392 | 0.34 | 1.08 | 26.8 | 5.4 |
| Adjusted Repeated Scan | 392 | 0.35 | 1.03 | 26.5 | 3.8 |
| Arm Circumference |  |  |  | ACZ >1 SD | ACZ >2 SD |
| Best-estimate Manual | 385 | 0.78 | 0.94 | 41.6 | 9.6 |
| Single Manual | 385 | 0.78 | 0.97 | 41.8 | 9.9 |
| Unadjusted Single Scan | 385 | 0.67 | 1.04 | 37.9 | 9.1 |
| Adjusted Single Scan | 385 | 0.77 | 1.03 | 41.6 | 10.6 |
| Adjusted Repeated Scan | 385 | 0.76 | 1.01 | 40.5 | 11.4 |
| *,***Significantly different from Best-estimate manual prevalence with Chi-Square at p<.10 and p<.01 | | | | | |
