# Supplementary material for: Accuracy and reliability of a low-cost, handheld 3D imaging system for child anthropometry
Source: PLoS One. 2018 Oct 24;13(10):e0205320. doi: 10.1371/journal.pone.0205320 (PMC6200231; doi:10.1371/journal.pone.0205320)
Supplement: S5 Table — Comparison to best-estimate manual measures among children 1–59.9 months of age. (DOCX) [file pone.0205320.s009.docx]

|  |  | Sensitivity | Specificity | ROC Area (Average of sensitivity and specificity) | ROC Area 95% CI | |
| --- | --- | --- | --- | --- | --- | --- |
|  |  |  |  |  | Lower Limit | Upper Limit |
| Stature (HAZ <-1 SD) | |  |  |  |  |  |
|  | Single Manual | 0.95 | 0.98 | 0.97 | 0.94 | 0.99 |
|  | Single Scan | 0.92 | 0.96 | 0.94 | 0.91 | 0.97 |
|  | Repeated Scan | 0.93 | 0.97 | 0.95 | 0.92 | 0.98 |
| Head Circumference (HCZ >1 SD) | | |  |  |  |  |
|  | Single Manual | 0.94 | 0.98 | 0.96 | 0.94 | 0.99 |
|  | Single Scan | 0.84 | 0.95 | 0.89 | 0.86 | 0.93 |
|  | Repeated Scan | 0.87 | 0.96 | 0.92 | 0.88 | 0.95 |
| Arm Circumference (ACZ>1 SD) | | |  |  |  |  |
|  | Single Manual | 0.93 | 0.95 | 0.94 | 0.91 | 0.96 |
|  | Single Scan | 0.91 | 0.94 | 0.93 | 0.90 | 0.95 |
|  | Repeated Scan | 0.93 | 0.96 | 0.95 | 0.92 | 0.97 |
