# Supplementary material for: Accuracy and reliability of a low-cost, handheld 3D imaging system for child anthropometry
Source: PLoS One. 2018 Oct 24;13(10):e0205320. doi: 10.1371/journal.pone.0205320 (PMC6200231; doi:10.1371/journal.pone.0205320)
Supplement: S1 Text — (DOCX) [file pone.0205320.s010.docx]

Supplementary methods, results and discussion

Conkle, J., Suchdev, P., Alexander, E., Flores-Ayala, R., Ramakrishnan, U., Martorell, R. Accuracy and reliability of a low-cost, handheld 3D imaging system for child anthropometry. Published online

This supplementary material has been provided by the authors to give readers additional information about their work.

**Methods**

**Test Methods**

For each scan of children two years of age and older an assistant positioned the child’s arms in one of three poses, and the measurer took a front and back scan of each arm pose by standing approximately two meters away from the child and holding the scanner at eye level (S2 Fig); for children less than two years of age we positioned arms away from the torso, but not necessarily in the three identified poses, and the measurer took scans by holding the scanner over the child. An individual scan captured 30 frames over one second. Multiple scans for one session provided redundancy in the case that movement blurred or hid a part of the child’s body. Children were measured undressed to their diaper or in skin-tight shorts/leotards provided by the study. Both members of a field team separately entered the child’s name, sex, race, ethnicity, and birth date from consent forms previously filled out by the child’s caretaker. AutoAnthro automatically uploaded data, including scans, to an online database. There were no reported adverse events from scans or manual measurements.

Anthropometrists received regular supervisor feedback on accuracy and reliability of manual measurements. Software included range checks to avoid data entry error for manual anthropometry, and automatic triggering of a third manual measurement based on Maximum Allowable Difference [1] to improve reliability. For demographic information we cleaned the data by checking for inconsistencies in double data entry. Following data collection, we processed scans to produce scan-derived measurements with no consideration of manual measurements. AutoAnthro had automated processing, which included automatically removing the image background and fitting the 3D point cloud obtained from each scan (S2 Fig) to an animator’s model, producing an intermediate result of six fitted models. For final measurements, AutoAnthro combined the six fitted models into a single, final model with best fit; and derived measurements based on points pre-selected once on the base model (Figures S3 and S4). We used two separate animator’s models: one for children under one month and another for children 1-59.9 months. To improve fitting efficiency, we used median measurement-for-age from WHO CGS [2] to automatically scale the animator’s model prior to fitting.

**Analysis Methods**

**Accuracy**

We assessed the accuracy of 3D imaging by comparing scan-derived measurements to the best-estimate from manual measurement. We calculated average bias with the following formula:

$AverageBias= \Sigma_{i=1}^{N}(M_{i1}- M_{iF1})/N$

, where $M_{i1}$ is the scan-derived measurement and $M_{iF1}$ is the manual best-estimate for each child.

We tested for differences in accuracy by caregiver-reported race and observed hairstyle using One Way Analysis of Variance (ANOVA). For comparing accuracy between races and hairstyles we used all-scan and best-estimate manual measurements to reduce variance and improve power. We coded hairstyle as a binary variable based on visual inspection of scans to identify children with protruding hair or hairstyles that could potentially affect scan-derived measurements. For race we defined three categories, namely Black, White, and Other; with the latter including Asian, Other, Multiple, and Not Reported. For ANOVA we tested the assumption of homogeneity of variances with Levene’s Test [3], and used Welch Test when variances were not homogenous [4]. We did not further disaggregate race by ethnicity because of sample size constraints.

**Reliability**

We calculated intra-observer technical error of measurement (TEM) for scan-derived and manual measurements with the following formula:

$TEMintra=\sqrt{\frac{\sum_{i=1}^{N} {(M_{i1}-M_{i2})}^{2}}{2*N}}$

, where N was the number of children and $M_{i1}M_{i2}$were the first and second measurements for one child (repeated measurements) by one observer. For inter-observer TEM we compared average measurements with the following formula:

$TEMinter= \sqrt{\frac{\Sigma_{i=1}^{N}{((\frac{M_{ij1}+ M_{ij2}}{2})-(\frac{M_{ik1}+ M_{ik2}}{2}))}^{2}}{2*N}}$

, where $M_{ij1} M_{ij2}$ were repeated measurements from one observer and $M_{ik1}M_{ik2}$ were repeated measurements from another observer of the same child. We also analyzed inter-observer TEM based on single measurements. For inter- and intra-observer TEM, we calculated relative TEM, or %TEM, by dividing TEM by the measurement mean and converting to a percent. Total TEM combined inter- and intra-observer TEM with the following formula:

$TotalTEM= \sqrt{{TEMintra}^{2}+ {TEMinter}^{2}}$

, where $TEMinter$ came from repeated measurements. We calculated an overall R, or Coefficient of Reliability, with the following formula from Ulijaszek [5]:

$R= 1- \frac{Total{TEM}^{2}}{{SD}^{2}}$

, where ${SD}^{2}$ was the pooled variance of the four measurements used to calculate Total TEM. We divided the sample into five age groups considering sample size for disaggregated analysis of reliability.

**Z-scores and Classification**

We calculated z-scores based on the 2006 WHO CGS using WHO SPSS 20 macros [6]. Anthropometric indices and nutritional status included in sample characteristics were based on best-estimate manual measurements. For additional analysis of z-scores and classification, we selected children 1-59.9 months of age to evaluate the animator model with sufficient sample size. To compare z-score means, standard deviations and prevalence we calculated z-scores from the best-estimate manual, single-manual, single-scan and repeated-scan. We tested for statistical significance of prevalence differences using Chi-Square and comparing to the prevalence from the best-estimate. We included both adjusted and unadjusted scan derived measurements, with adjustment made by subtracting or adding the average bias found in our analysis of accuracy from or to each observation. We evaluated individual level classification with Stata’s *diagt* module, reporting sensitivity and specificity for stature-, head circumference-, and arm circumference-for-age below or above 1 standard deviation. For individual level classification we considered the best-estimate “true” nutritional status. We selected z-score SD cutoffs based on having an adequate percentage of the sample selected by the cutoff for meaningful comparisons of z-scores and classification. We did not have sufficient sample size to analyze differences in z-scores and classification for newborns, or to analyze individual level classification at lower cutoffs, such as <-2 SD.

**Results**

**Z-Scores and Classification Results**

For scan-derived measurements, we calculated z-scores based on both unadjusted and adjusted measurements, with adjustments made to remove systematic inaccuracy by subtracting or adding the average bias in Figure 2 from or to each observation. All height-for-age z-scores (HAZ) and head circumference-for-age z-scores (HCZ) were biologically plausible. One child was flagged for an implausibly high arm-circumference-for-age z-score (ACZ), and this same child was flagged by both manual and scan-derived measurements (with or without adjustment). Mean z-score values and percentages below or above SD cutoffs presented in S4 Table show that, as expected, the accuracy adjustments helped to reduce the difference between scan-derived and best-estimate results. Unadjusted, single-scan HC mean z-score was 0.2 higher than the best-estimate mean z-score, and the percentages above 1 SD and 2 SD were overestimated by five percentage points; after adjustment, single-scan HC and best-estimate mean z-scores were nearly the same and percentages differed by less than two percentage points. Percentages based on adjusted, scan-derived measurements from single or repeated sessions were within two percentage points of percentages based on best-estimate manual measurements for all measures. Z-score SDs for single-scan were higher than z-score SDs from single-manual, which is the effect of less reliability seen in Figure 3. Using repeated-scan brought the z-score SD closer to the level of manual measurements.

For individual level agreement we analyzed sensitivity and specificity of HAZ <-1 SD, HCZ>1 SD, and ACZ>1 SD among children 1-59.9 months with the best-estimate considered “true” nutritional status. Sensitivity for stature measured the probability that a child’s HAZ based on the adjusted scan-derived measurement was <-1 SD given that HAZ based on the best-estimate was <-1 SD. Stature sensitivity was 0.95, 0.92, and 0.93 for single-manual, single-scan, and repeated-scan respectively; indicating that out of 100 children identified as HAZ <-1 SD by the best-estimate, 95, 92 and 93 children would also be identified by single-manual, single-scan, and repeated-scan respectively (S5 Table). Stature specificity was also high; out of 100 children not identified as HAZ <-1 SD, 98, 96, and 97 of those children would also not be identified as having HAZ <-1 SD by single-manual, single-scan, and repeated-scan respectively. For stature and arm circumference, sensitivity and specificity of single- and repeated- scan was excellent and performed nearly as well as single-manual. For scan-derived head circumference specificity was excellent, but sensitivity was 0.84 and 0.87 for measurements based on single-scan and repeated-scan respectively (S5 Table).

**Discussion**

**Single versus Repeated Scan Reliability**

We designed the 3D imaging system to derive measurements based on a single scan session. In this study we found that single-scan was less reliable than single manual measurements, but that averaging two measurements from two scan sessions (repeated-scan) improved TEM and achieved reliability similar to repeated manual measurements. Repeated-scan achieved excellent reliability and single-scan was less reliable, but the difference between single and repeated scan reliability was not large (indicated by difference between inter- and intra-observer TEMs in S3 Table. Furthermore, the differences in z-score SDs and percentages below or above SD cutoffs for single- and repeated-scan were small and statistically insignificant. Additional research that is adequately powered to detect small differences in prevalence is needed to determine if the protocol for deriving measurements from 3D imaging should be changed from using a single-scan to repeated-scan. We believe the use of repeated scans is feasible and would not be overly burdensome for the anthropometrist or child because repeating scans would only add approximately one minute to measurement time. We will be able to quantify the effect of a change in protocol on the time required for collecting child anthropometry in a time-motion study that is underway to compare 3D imaging with manual measurements.

We did not have maximum allowable difference triggers to improve reliability of 3D imaging because scan-derived measurements are not immediately available, and so did not use triggered, third manual measurements for calculating inter-observer TEM in this study. However, we compared inter-observer TEM results from this study to a previous publication where we did use the third manual measurements [7] and we found that triggers did little to improve our manual measurement reliability; the reliability of repeated-scan is similar to manual measurements with or without the use of maximum allowable difference triggers for manual measurements.

**The Effect of Reliability and Accuracy on Monitoring and Classification**

We were able to obtain accurate results from 3D imaging that yielded mean z-scores and z-score standard deviations similar to manual measurements, but only after making adjustments to remove systematic inaccuracy. After adjustment, the percentages of the population below or above z-score cutoffs for HAZ, HCZ, and ACZ according to 3D imaging were similar to manual measurements, and there was a high probability that a child identified as being below or above z-score cutoffs by manual measurement would be identified the same way by 3D imaging. Anthropometry is used to classify the nutritional status of individuals and populations. There is no single standard for judging the adequacy of child anthropometry reliability and accuracy [7], but ultimately if a method correctly classifies nutritional status and can detect changes in nutritional status over time, reliability and accuracy of that method should be considered acceptable. In our study 3D imaging was reliable enough to be considered a good method for collecting child anthropometry, but systematic inaccuracy caused misclassification.

We showed that 3D imaging produced reliable measurements; we also need to consider how this reliability would affect the quality of anthropometric data outside of a research setting. Most large-scale surveys do not take repeated measurements and so cannot be analyzed for reliability, but reliability is directly related to z-score SD. Reliability is a metric of random error, and as random error increases z-score SD increases. If z-score SD is too high, there may be overdispersion, which can cause overestimation of prevalence below or above z-score cutoffs. High quality surveys have z-score SD between 0.9-1.1 [8, 9]; in this study 3D imaging z-score SD was within 0.9-1.1 for all measures. An evaluation of the quality of 52 DHS found that 1 out of 52 had HAZ SD between 0.9-1.1, and 30 out of 52 had HAZ SD above 1.5 [10]. A SD of 1.5 can lead to overestimation of prevalence. A recent study found that DHS and MICS carried out in Western and Central Africa from 1990-2012 may have overestimated the prevalence of stunting (HAZ <-2 SD) by approximately 10 percentage points on average [9]. Overdispersion from poor quality anthropometry can also result in overestimation of overweight and obesity. Poor quality anthropometric data is common in large-scale surveys, and quality is variable between countries and between surveys in the same country; making it difficult to meaningfully compare countries or analyze trends over time. The reliability of 3D imaging and manual anthropometry in BINA was good enough to make meaningful comparisons between countries and over time. Our reliability findings also indicate that 3D imaging can be used for growth monitoring. Considering a well check schedule of visits at 1, 2, 4, 6, 9, 12, 15, 18 and 24 months; the differences in stature between visits is 3.2-5.5 cm according to the 2006 WHO CGS median. The inter-observer TEM for repeated-scan indicates that repeated measurements from 3D imaging can be within 1.5 cm 99.9% of the time — 3D imaging random error would not cause overlapping estimates in growth monitoring.

We measured accuracy with average bias, a metric of systematic bias. In the MGRS average bias was considered acceptable if it was within ±2.8 times the expert intra-observer TEM [11]; based on MGRS criteria, 3D imaging accuracy was acceptable for all measures in our study. However, our findings indicate that the criteria used to assess average bias for the 2006 WHO CGS may be too lenient for the purposes of our study. For head circumference the acceptable average bias was set at ±0.34 cm in the MGRS [11]. In our study the average bias for head circumference from scan-derived measurements among children 1-59.9 months of age was well within the acceptable range at 0.26, but our HC average bias led to a statistically significant five percentage point overestimation of the percent of the population above 1 SD and 2 SD when compared to manual measurement (Table 2). Since 3D imaging produced measurements that were essentially as reliable as manual, we can conclude that the differences in prevalence are due to inaccuracy, showing that HC average bias of 0.26 is likely not acceptable. The acceptability of average bias should be based on producing similar prevalence estimates and avoiding misclassification of individuals. For prevalence estimates there is no single criteria for how close is close enough, but we can consider how prevalence is used to posit reasonable criteria. The WHO cutoff for a moderate public health problem for wasting is 5%, which is approximately 2.5 percentage points from what is expected in a healthy population. It is reasonable to say that differences in prevalence between an index test and a reference standard cannot exceed 2.5 percentage points for child anthropometry. From another perspective, differences between the two methods should ideally not be statistically detectable in a common, large scale-survey; and should allow for meaningful program evaluation, which would put the level closer to 1 percentage point. A typical program goal may be to reduce stunting by one to two percentage points annually over three to five years. A program effect may be statistically masked by error over two percentage points. After adjustment, prevalence differences between repeated-scan and best-estimate in our study were less than one percentage point for stature and head circumference, and less than two percentage points for MUAC; which is probably good enough for common uses of prevalence estimates. For individual classification sensitivity and specificity above 0.90 is generally considered excellent. We were able to achieve excellent classification at the individual level, but only after adjusting measurements to remove systematic bias.

**Bias Hypotheses**

3D imaging overestimated head circumference, while underestimating MUAC. Consideration of the protocol for manual measurements provides a hypothesis for the different directions of average bias. When measuring head circumference, anthropometrists were instructed to apply enough tension to compress the hair and underlying soft tissue. For MUAC, the measuring tape was meant to be flush with the skin without any compression. 3D imaging may have overestimated head circumference because compression of soft tissue was not taken into account during calibration. If compression is the source of bias for head circumference, we may expect no average bias for MUAC because there is no compression for MUAC. Interestingly, for MUAC ≥ 16.7 cm we found essentially no systematic bias, and it was only among smaller children (MUAC 9.6-15.1 cm) that we found substantial underestimation. It is possible that underestimation by 3D imaging for MUAC was caused by manual measurement error. It can be more difficult to measure MUAC on younger children because increased adiposity along with decreased cooperation make it more difficult to maintain the measuring tape flush against the skin. It is possible that BINA anthropometrists systematically left small gaps between the skin and measuring tape when measuring MUAC of younger children in an attempt to avoid the mistake of compressing the skin; compression is a typical source of bias when MUAC is used in screening and surveys. In our study, manual MUAC measurement may have been less accurate than 3D imaging for young children. Future studies should consider the use of a wider MUAC measuring tape, which was recently shown to be more reliable than current measuring tapes [12].

The protocol for manually measuring head circumference may also help to explain another finding: 3D imaging was better at selecting the same children above or below SD cutoffs for stature and MUAC than it was for HC. The protocol for measuring manual HC calls for shifting the measuring tape up and down to select the largest circumference; there is no fixed position. The current 3D imaging system is based on measuring fixed, pre-specified points; and does not account for being able to shift the measuring tape up and down. To achieve excellent sensitivity 3D imaging system software may have to be developed to identify the largest circumference and not just rely on pre-specified points.

When processing scans we returned the final 3D model to a “neutral” position to take stature measurements. Comparing the “neutral” position to WHO measuring protocol, we did not take into account the Frankfort Plane and the back of the head was not aligned with the heels, buttocks, and back of shoulders. It is not clear what effect model positioning had on the accuracy of scan-derived stature and if positioning is a reasonable hypothesis for overestimation. Another possibility for overestimation is that 3D imaging did not adequately take into account hair compression that occurs when taking manual stature measurements.

**Study Limitations Related to Automation**

Per study protocol children were required to adopt fixed positions when taking scans, but anthropometrists could not touch the child because touching impaired the ability to process the scans into measurements. During pretesting we discovered that an anthropometrist could help to put the child into position if both the measurer and the child held a common object, such as a large, plastic spoon. However, for some children this approach did not work due to lack of cooperation, thus we further altered the protocol by allowing anthropometrists to take excess scans. When anthropometrists took extra scans, they then selected the 12 best scans for each child and deleted the rest. Another departure from the original protocol was that anthropometrists could not always take scans in the specified sequence of orientation: front and then back. With many children anthropometrists had to be opportunistic and take scans of a child when they were still, ignoring their orientation. For processing scans we had to manually code orientation after data collection. Processing scans was initially designed to be fully automated; the manual input to delete excess scans and define the child’s orientation make our findings less generalizable because there is more potential for human error, and we do not know if the number of scans taken per child affected accuracy or reliability. Further development of the 3D imaging system scanning and processing software is needed to achieve full automation.

**References**

1. de Onis M, Onyango AW, Van den Broeck J, Chumlea WC, Martorell R. Measurement and standardization protocols for anthropometry used in the construction of a new international growth reference. Food Nutr Bull. 2004;25(1 Suppl):S27-36. PubMed PMID: 15069917.

2. De Onis M, World Health Organization. Dept. of Nutrition for Health and Development. WHO child growth standards : length/height-for-age, weight-for-age, weight-for-length, weight -for-height and body mass index-for-age : methods and development. Geneva: World Health Organization; 2006.

3. Brown MB, Forsythe AB. Robust Tests for the Equality of Variances. Journal of the American Statistical Association. 1974;69(346):364-7. doi: 10.1080/01621459.1974.10482955.

4. Welch BL. On the Comparison of Several Mean Values: An Alternative Approach. Biometrika. 1951;38(3/4):330-6. doi: 10.2307/2332579.

5. Ulijaszek SJ, Kerr DA. Anthropometric measurement error and the assessment of nutritional status. The British journal of nutrition. 1999;82(3):165-77. Epub 2000/02/03. PubMed PMID: 10655963.

6. WHO. WHO Anthro and macros Geneva: WHO; 2015. Available from: http://www.who.int/childgrowth/software/en/.

7. Conkle J, Ramakrishnan, U., Suchdev, P., Flores-Ayala, R., Martorell, R. Improving the quality of child anthrompometry: Manual anthropometry in the Body Imaging for Nutritional Assessment Study (BINA). PloS one. 2017;12(12). doi: [10.1371/journal.pone.0189332](https://doi.org/10.1371/journal.pone.0189332)

8. SMART Technical Advisory Group. The SMART Plausibility Check for Anthropometry. Toronto: Action Against Hunger - Canada; 2015.

9. Grellety E, Golden MH. The Effect of Random Error on Diagnostic Accuracy Illustrated with the Anthropometric Diagnosis of Malnutrition. PloS one. 2016;11(12):e0168585. doi: 10.1371/journal.pone.0168585.

10. Assaf S, Kothari, M., Pullum, T. An Assessment of the Quality of DHS Anthropometric Data, 2005-2014. DHS Methodological Reports No 16. Rockville, MD, USA: ICF International; 2015.

11. WHO Multicentre Growth Reference Study Group. Reliability of anthropometric measurements in the WHO Multicentre Growth Reference Study. Acta Paediatr Suppl. 2006;450:38-46. PubMed PMID: 16817677.

12. Hess SY, Hinnouho GM, Barffour MA, Bounheuang B, Arnold CD, Bell D, et al. First Field Test of an Innovative, Wider Tape to Measure Mid-Upper Arm Circumference in Young Laotian Children. Food Nutr Bull. 2018;39(1):28-38. Epub 2017/12/21. doi: 10.1177/0379572117742502. PubMed PMID: 29258337.
